# Medical and Philosophical Intelligence

**Published:** 1818-03

**Authors:** 


### MEDICAL AND PHILOSOPHICAL INTELLIGENCE.

*ROYAL SOCIETY.*—St. Andrew's Day falling this year on a Sunday, the Royal Society held their annual meeting December 1st, at their apartments in Somerset-place, when the president, the Right Honourable Sir JOSEPH BANKS, Bart, G.C.B., after a very able speech on the determination of an invariable Standard of Linear Measure, presented, in the name of the society, the gold medal, called the Sir Godfrey Copley's medal, to Captain HENRY KATER, for his Experiments for determining the Length of the Pendulum vibrating Seconds in the Latitude of London. The society afterwards proceeded to the choice of a council and officers for the year ensuing; when, on examination, it appeared that the following gentlemen were elected:—

*Of the Old Council.*—The Right Honourable Sir Joseph Banks, G.C.B.; William Thomas Brande, Esq.; Samuel Goodenough, Lord Bishop of Carlisle; Taylor Combe, Esq.; Sir Humphry Davy, Knt. LL.D.; Sir Everard Home, Bart.; Samuel Lysons, Esq.; George, Earl of Morton; John Pond, Esq. Astronomer-royal; William Hyde Wollaston, M.D.; Thomas Young, M.D.

*New Council.*—George, Earl of Aberdeen; Davies Gilbert, Esq. M.P.; Charles Hatchett, Esq.; Captain Henry Kater; William Howley, Lord Bishop of London; Right Honourable Charles Long, M.P.; John Reeves, Esq.; Richard Anthony Salisbury, Esq.; Edward Adolphus, Duke of Somerset; Glocester Wilson, Esq.

OFFICERS.

*President.*—Right Honourable Sir Joseph Banks, Bart. G.C.B.

*Treasurer.*—Samuel Lysons, Esq.

*Secretaries.*—William Thomas Brande, Esq.; Taylor Combe, Esq.

By a paper read before the Society, it appears Sir Everard Home is returning to those microscopical observations on the blood, which his master undervalued, perhaps, too much. We cannot, however, help remarking, that, if Mr. Bauer's microscopes magnify so greatly, they must increase the uncertainty in the same ratio. We are told, that 2,560,000 globules of human blood, when enveloped in their colouring matter (which Sir E. conceives to be something superadded to their proper substance) would be required to cover a square inch. He supposes that the globules possess a regularly-organized structure: they were observed to range themselves in lines, which, when examined and compared with the muscular fibre, under high magnifying powers, led to the conclusion, that these particles are the constituents of the fibre. The blood in coagulating assumes a tubular texture, an effect produced by the extrication of gas during the coagulation.

Dec. 11.—A Memoir, by Captain Burney, was read, on the Geography of the North-eastern Part of Asia, examining the question, Whether Asia and America are united? which he concludes in the affirmative: of course, that no north-west passage, so often sought for, exists.

Dec. 18.—A paper, by James Smithson, Esq. was read, on the Colouring Properties of Certain Matters. Among the substances examined were litmus, the colouring matter of violet, the blue paper employed to cover loaf-sugar, the blue of hyacinth, the mulberry, the corn-poppy, and sap green. Mr. Smithson thinks it probable, that some vegetable colours may be produced by combining their principles.

[We shall take an early opportunity of an Analysis of the present controversy concerning chlorine.]

---

*Royal College of Surgeons.—Hunterian Oration.*—The venerable appearance of Sir D. Dundas, little known, but by name, to most of the London practitioners, produced a sudden sensation as he entered the Theatre. Though his voice is weak, yet, such is the happy structure of the place, that every sound was heard in the most distant part. He began by an extempore apology for his not being accustomed to public speaking, and lamenting that, on the present occasion, this office was imposed on him by the death of Sir James Earle, in terms which did honour to his feelings and to the memory of the deceased. A short exordium followed on the services of the late Mr. Hunter, which could be best illustrated by tracing the history of surgery before his time. Nothing particularly new could be expected in such a sketch, but whatever was most striking was brought forward in the most interesting manner. Ambrose Parée furnished a very valuable source of anecdotes, particularly on the treatment of gun-shot wounds. The French Royal Academy of Surgery was properly noticed. In England, Wiseman, Sharp, Pott, and a few others, were introduced with much propriety and justice. The mention of Mr. Pott again introduced Sir James Earle, his disciple, his son-in-law, and his meritorious successor. He dwelt slightly on the improvements he had added to surgery, particularly in the operation for the hydrocele,—before his time truly formidable, but since reduced to the greatest simplicity.

Mr. Hunter he considered as forming the foundation of surgical pathology. His account of all the properties of the blood, which could only exist from its living principle; his experiments to prove that solidity is not essential to life, and that even the fluidity of the blood was only to facilitate its circulation, were dwelt upon as far as was necessary before an audience, most of whom were supposed to be well acquainted with the subject. His practical improvements on gun-shot wounds,—on the treatment of broken tendons,—and, above all, his operation for aneurism, the result of profound reasoning, from an accurate estimate of the unknown powers of the animal system by those which he had already ascertained,—formed a splendid and well-rounded period.

It was natural that one, who has so long and so deservedly basked in the sunshine of royalty, should take this opportunity of acknowledging the services which the College had received from that source. To Henry VIII. the Surgeons owed the first separation from the Barbers. By his present Majesty, they were raised from a corporation to a Royal College. By the liberality of the same illustrious personage, and by his recommendation to Parliament, they were enabled to purchase the Hunterian Collection, and to erect the present building for its reception, as well as for those public lectures by which it was illustrated, and in which the present just encomium was pronounced.

The account of Mr. Hunter's theory of the vitality of the blood, and of his experiments on animal heat, by which he proved that it could neither be the effect of any chemical combination in the lungs, nor of the brain and nerves, with various other discoveries, were neatly introduced; and, with a few affectionate wishes for the perpetuity of the College, the oration closed. Every part was well attended to, and gave satisfaction to every hearer.

---

*Surgeon's Bill.—Preamble:* "Whereas ignorant and incapable persons are not restrained by law from practising surgery; whereby the health of great numbers of persons is much injured, and the lives of many destroyed;"

It shall not be lawful for persons to practise surgery, unless examined by the College of Surgeons, and receiving a diploma. The College of Surgeons includes any one of the three kingdoms.

Army-surgeons, having been duly examined, are permitted to practise.

For extending the provisions of Irish Acts to members of English and Scotch colleges: "Be it therefore enacted, that the members of the Royal College of Surgeons in London, and of the Royal College of Surgeons in Edinburgh, and of any other College of Surgeons which may hereafter be incorporated as aforesaid, shall be eligible to all the offices and appointments mentioned in the first recited Act, and shall be entitled to all benefits and advantages given and intended by the second recited Act, on the production of the diplomas or testimonials under the seal of their respective colleges, in the same manner as the members of the Royal College of Surgeons in Ireland have been since the passing of the said recited Acts.

"And whereas surgical knowledge is frequently required in the practice of midwifery, and it is expedient that male persons so practising should be qualified to render surgical aid; Be it therefore enacted, That, from and after ——, it shall not be lawful for any male person to practise midwifery, unless he shall have obtained a diploma or testimonial, as aforesaid, of his knowledge and ability to practise surgery, under the seal of one of the said Royal Colleges, or of any other College of Surgeons which may hereafter be incorporated as aforesaid; or unless he shall have obtained, as aforesaid, a testimonial of qualification as a principal surgeon in the army or navy, and shall have actually served in that capacity; or unless he shall have obtained the degree of Doctor or Bachelor of Medicine from an University in the United Kingdom.

"Provided always, and be it further enacted, That nothing in this Act contained shall be deemed or taken to extend to any person resident in Great Britain or Ireland, and actually practising surgery or midwifery at the time of the ——; but that every such person may continue to practise surgery or midwifery respectively, so far as any such person lawfully might have done if this Act had not been passed.

"Provided always, and be it further enacted, That nothing in this Act contained shall extend, or be construed to extend, to lessen, prejudice, or defeat, or in anywise to interfere with any of the rights, authorities, privileges, or immunities, heretofore conferred upon, vested in, or legally exercised and enjoyed, by any of the Universities in the United Kingdom, or by any of the Colleges of Physicians in the United Kingdom, or upon, in, or by the Faculty of Physicians and Surgeons of Glasgow, or by the master, wardens, and Society of the art and mystery of Apothecaries of the city of London; but that the members of the said Universities, and of the said Colleges of Physicians, and of the said Faculty of Glasgow, and of the said Society of Apothecaries respectively, shall have and enjoy all such rights, authorities, privileges, and immunities, in as full, ample, and beneficial a manner, to all intents and purposes, as they might have done before the ——, and in case the same had never been passed."

---

*Committee Room, Crown and Anchor Tavern, February 4th,* 1818.—At a Meeting of the Committee of the Associated Apothecaries and Surgeon Apothecaries of England and Wales, Mr. Parkinson in the Chair:—The committee having endeavoured to ascertain the most appropriate means of securing the objects of this association, and having resolved on the measures at present to be adopted, further agreed unanimously to the following resolutions:—

1st. That it is exceedingly gratifying to observe, that the public health and the respectability of the profession are much protected by the Act of Parliament originating with the former committee of this association, and procured through the exertions of the Society of Apothecaries.

2d. That the public advertisements of the Society of Apothecaries, declaring their determination to prosecute any person practising as an apothecary, in violation of the said Act, will, it is presumed, have considerable influence in preventing the intrusion of unqualified pretenders into the practice of medicine.

3rd. That this committee regards with great satisfaction, the liberal manner in which public lectures have been instituted by the Royal College of Surgeons, and by the Society of Apothecaries; convinced that the public welfare, as well as of the best interests of this association, must be promoted by the extension of professional knowledge.

4th. That the preceding resolutions be transmitted to the several Medical Journals.

---

*To the Editors of the London Medical and Physical Journal.*

Gentlemen,—Under the head of an article of intelligence in your last Number, is the case of a deaf and dumb boy. With respect to that case, you will find it detailed in my Treatise on the Physiology and Diseases of the Ear, page 90. The boy has been seen by several respectable medical characters of the metropolis, his name is Thomas Hamilton, he resides with his parents, No. 12, King-street, Drury-lane, where you may see him.

I am, Gentlemen, your obedient Servant,

2, *Soho-square;* J. H. Curtis,
*Feb.* 10, 1818. Surgeon to the Royal Dispensary.

---

*Case of Fractured Humerus, occasioned by the mere Effort of Muscular Power;* by M. Odienne.

Though most modern surgeons, (and particularly Richerand,) deny the possibility of fractures by the mere effort of muscular contraction, it is not less certain that these fractures may happen in the long bones, though an exact knowledge of anatomy and the animal mechanism, seem to shew its impossibility; and the most brilliant theories must submit to facts.

Lewis Laire, of Favril, a day-labourer, twenty years old, of a lymphatico sanguine temperament, in very good health, was playing (the 24th of April, 1814,) with several young men of his age, and threw a stone with violence at one of them, who was leaving the company. At the moment the stone left his hand, a sharp crack was heard in the lower part of the arm, which he immediately let fall, on account of the violent pain he felt in one spot, which in the course of two hours swelled very much.

He came to consult me directly, begging me to give him relief as soon as possible. After having uncovered the part, I found the arm bent, concave internally and convex behind. He could not make the slightest motion of the fore-arm, without the most violent pain, three inches and a half above the radio-cubital articulation near the middle of the length of the anterior brachial muscle, where the fracture was, and where the two extremities of the bone made a very distinct crepitus. On moving the hand in the direction of the humerus, and its posterior part, a very distinct inequality was perceptible in the spot described. In short, every thing concurred to leave no doubt of the existence of the fracture, which was easily reduced, and after its consolidation, the bone remained a long time in that part larger than in the natural state.

---

*Observations on the Application of Fire in the Cure of Sciatica;* by M. Potet, Surgeon, and Member of the Society, at Evreux.

*First Observation.*—Godard, of the parish of Villalet, thirty-six old, of a strong constitution, had walked with crutches for two or three years, on account of a sciatica in the right leg.

I advised a train of gunpowder to be burnt on the lame leg, from the external malleolus to the head of the femur; to which he consented.

Having placed him in a proper position, I set fire to three trains of gunpowder, one after the other, which only produced little vesicles that did not suppurate: notwithstanding, four days after the application of this new moxa, he walked without crutches, though he used a stick to go and work in his farm. It is now four years since this sort of cure was effected.

*Second Observation.*—The widow Milard, from the parish of Nogent-le-Ser, about sixty years old, was attacked with a sciatica, that extended to the middle of her leg; I burnt only two trains of gunpowder on the affected leg and thigh; but I had the satisfaction to see her go home on foot: she assured me that the fire took off the pain directly, and nothing remained but the smart of the burn, which was nothing in comparison of what she had suffered before.

*Third Observation.*—Eighteen months ago, I was desired to visit Faucher, of the parish of Huest, a thrasher, about forty years old, of a weak constitution, who had been confined to bed for two months, with a rheumatic pain in both legs, which had resisted the treatment of a practising apothecary.

I burnt two trains of powder on the leg and thigh which suffered most; he would not try a third: nevertheless, in eight days he walked without pain, and resumed his business of thrasher, as soon as he could get employment, and now works at St. André. He had felt some pains in the leg that was not fired, and desired me to burn some powder on that, but the pains ceased spontaneously.

Did the fire destroy the humour which caused the pain of the sciatica?

Might not this new moxa be useful in the bite of mad animals?

I leave these questions to be decided by physicians.

---

*Note on a Female Dwarf, Seven Years Old, with the Proportions nearly of a New-born Child;* by M. Beclard. Read to the Faculty of Medicine of Paris.

The name of this little girl is Anna Barbara Schreyerin. Her father is of a middling size, and she has three sisters and a brother of the ordinary size of their age. She was born at the full term; and it is said, that at the time of her birth she was eight inches long, and weighed a pound and a half; which is the common length of a fœtus in the fourth month of conception, and its weight at five months and a half.

She was born the 31st of October, 1810, and consequently was seven years old last October. Her height is twenty-one inches and a half, and her weight eight pounds and a half: which is the common length and weight of a child a month old.

But the proportions of her body are different from those of a fœtus; from the sole of the foot to the eminence above the pubis, she is nine inches long, and from the sole of the foot to the navel, ten inches; and from the summit to the eminence above the pubis, twelve inches and a half. Thus, the middle of the body answers nearly to the re-union of the one-third superior, with the two-thirds inferior parts of the space comprised between the navel and the eminence of the pubis; while in the full-grown fœtus the middle of the body is nearly between the navel and the superior extremity of the sternum.

The diameters of the thorax, pelvis, and head, measured exactly with a compass for thickness, are in general a tenth larger than those of the same parts in a new-born child.

The bones are well formed. The teeth are sufficiently indicative of the age of seven years; and, the first permanent teeth are come forth in both jaws. Several temporary teeth have fallen; and the first permanent superior right incisor is appearing through the gum.

The muscles are sufficiently firm, and well drawn under the skin, though almost void of the subjacent adipose tissue. The muscular action sufficiently strong, and in continual exercise, is irregular, and even shows a myotybia pretty well marked.

The senses are regular in their conformation and action, except the eyes, which are myops; and of which the left is turned inwards.

The nutritive functions are regular in their exercise; but it is impossible to count the beating of the arteries for a minute, on account of her impatience and continual motion.

It is useless to repeat, after all the modern writers on the natural history of the human species, that there is no race of dwarfs any more than of giants, and that the natural stature of man is confined within pretty narrow limits.

Or, that the different dwarfs which have been observed at several times, belonged even to dwarf families; but appeared as exceptions in the midst of families of which the other individuals were of the ordinary human stature.

Or, in short, that most of these dwarfs were idiots; that the intelligence of the others was very confined, and that almost all died of decrepitude before thirty; as if the duration of their life had been measured by their stature, or rather on the whole of their confined organization.

One of us had occasion to see a case, ten years ago, which may serve as a counterpart to this one presented to the society; it was a man twenty years old, not yet arrived at the age of puberty; whose growth was not yet completed, but who was already six feet ten inches high.

This particular fact, which the society has had an opportunity of observing, seems sufficiently curious to be modelled in wax, and placed in the museum of the school, by the figure of Bebé.

---

*Memoir of Sir Richard Croft.*—Sir Richard Croft, Bart. M.D. served an apprenticeship to Mr. Chavasse, an apothecary, at Burton-upon-Trent, where he betrayed marks of a comprehensive mind. On the expiration of the term of his servitude, his parents sent him to London, to complete his medical education. He entered as a student at St. Batholomew's about the year 1801 or 2, when the celebrated Pott and the late Dr. David Pitcairn were among the medical officers of that grand institution. He had in the number of his fellow students, the late Dr. Willan, Dr. Stokes, of Chesterfield, and Dr. Adams. He became also a pupil of the celebrated Dr. Hunter; and, by the recommendation of Dr. Baillie, a fellow pupil, boarded and lodged with Dr. Denman, an apothecary, then living in Queen-street, Golden-square (his house being contiguous to Windmill-street, Mr. D. contrived to improve his income by boarding and lodging the pupils of Dr. Hunter.) In this family, he and his friend Baillie met with that kind of rational amusement from the society of Denman and his twin daughters, which studious characters require to relax their minds, in order to enable them more effectually to prosecute their inquiries. The Duchess of Newcastle, who was then pregnant, and in a bad state of health, being advised by Hunter to go to Portugal, engaged Dr. Denman, on the recommendation of Dr. Hunter, to accompany her, chiefly for the purpose of superintending her labour. Her Grace having a good time, and the climate having greatly improved her general health, she and the doctor returned to London. Soon after their arrival, Hunter discharged his debt to nature, and her Grace exerted all her interest to secure for Dr. Denman Hunter's midwifery practice. Dr. Denman finding, that through her Grace's interest, he should be established as the fashionable accoucheur in London, relinquished his shop and boarding-house, purchased a diploma, and started as a physician-accoucheur. To give an importance to his professional character, he commenced lectures on the science of midwifery, and the diseases of children, for all which he was well qualified. Fortunate as this occurrence was for Dr. Denman, it was no less so for the medical profession; for it was the means of bringing forward talents which would otherwise have been lost to the world; and, in this metropolis, many are the practitioners who obtain a scanty livelihood by the trade of an apothecary, who only want the same good fortune to bring them into notice. Dr. Denman, by his lectures, proved himself to be a man of strong intellect, great ingenuity, and scientific attainments; and to him we are indebted for the best general treatise on midwifery that has appeared in this or any other country. Mr. Croft commenced his career as surgeon, apothecary, and man-midwife, at Tudbury, where a predilection for the sports of the field introduced him to Lord Vernon. From Tudbury he went to Oxford, which he quitted for London. Dr. Denman being now in great practice, Mr. Croft and Dr. Baillie renewed their acquaintance with his daughters, whom they soon afterwards conducted to the altar. It fortunately happened about this time, that the Duchess of Devonshire was approaching the period of her accouchment in Paris, and sent for Dr. Denman to attend her on that occasion. The doctor's other engagements not permitting his absence, he recommended Mr. Croft, whose success secured an universally favorable reception among the ladies of rank in London. Denman having acquired an independence, gradually withdrew from the fatigue of practice, in order to introduce his sons-in-law; and this he managed with so much dexterity, that Mr. Croft in a short time succeeded to the whole of his practice—probably in consequence of Dr. Denman's having intimated that he would give him his attendance in cases of difficulty. Dr. Baillie being also the nephew of Hunter, a powerful interest was thus formed, sufficient to secure the best practice in the metropolis for the sons-in-law of Denman.

Sir Richard Croft succeeded to the Baronetcy on the death of Sir Herbert Croft, a gentleman well known in the literary world. Sir Herbert had been some years at the bar, which he quitted for the church. On the lamentable affair of Miss Ray and Hackman, he related the story in a work, entitled Love and Madness, which formed an interesting miscellany, though relating principally to events of a melancholy nature. When Dr. Johnson was writing the Lives of the British Poets, not being sufficiently acquainted with the biography of Dr. Young, he applied to Mr. Herbert Croft, (not then a baronet) to supply the deficiency. Mr. H. Croft wrote the article requested, and has imitated Dr. Johnson's style so well, as to give a uniformity to the whole of that treasure of biography. On the death of Dr. Johnson, Sir Herbert was preparing to publish a new edition of his dictionary, with the addition of many thousand words.

The above account is copied from one of the daily prints. For the greater part we can vouch. We are not sure that Sir Richard ever purchased a diploma, but it is certain that he did not assume any title before his baronetcy, though he was generally, by a kind of courtesy, called Dr. Croft.

Sir Richard was a man of an excellent heart, a good sound understanding, and great diligence. His texture seemed delicate, which evinced itself in his figure, countenance, and articulation. His manners were pleasant, his conversation full of anecdote, which became often the more interesting from the slow manner in which he was obliged to speak.

His life affords a very striking instance of human uncertainty. Elevated to the highest pinnacle of professional importance, and expecting to bring into the world the first of a new race of kings, we see him instantly plunged into a gulph of distress, from which he saw no means of extracting himself but one, the recollection of which strikes us with horror.

It would be perfectly absurd to suspect any want of professional knowledge in one educated regularly, and having all the advantages of his father-in-law's experience. But most of his surviving brethren now regret, that he suffered the whole responsibility to rest with himself. Yet who reflected so far before the fatal issue was announced?

We have heard an anecdote highly to the honor of Dr. Denman and his son-in-law. On Mr. Croft's return to London, (as the above account states), he renewed his acquaintance with Dr. Denman, whose daughters had then arrived at the most interesting age. Educated as they had been, their society could not be indifferent to a young man who recollected them at a more tender age. The effect produced on Croft was such, that he conceived it prudent to impose some restraint on his visits: this was observed by Dr. D. with some surprise; and, when he was aftewards made acquainted with the cause, he desired no such scruples might interfere. Considering the uncertainty of Mr. Croft's prospects at that time, such a conduct marks a liberality untainted by ambition or any selfish prospects, and evinces also the good opinion Dr. D. entertained of his future son-in-law.

---

Professor Bianchi, of Pisa, has published a number of experiments to show the uncertainty of Tincture of Turnsole, and other vegetables, as chemical tests.

---

Mr. Curtis has in the press, an Introductory Lecture to his Course, on the Anatomy, Physiology, and Diseases of the Ear, as delivered at the Royal Dispensary.

---

Mr. Clarke will commence his next Course of Lectures on Midwifery, and the Diseases of Women and Children, on Friday, March the 20th.

### REPORT OF DISEASES.

Every returning month, by calling on us to reflect on the past, convinces us of the necessity of recurring to the manner in which Sydenham attended to the changes in the constitution of the atmosphere, and their effect on the animal frame. Whoever studies that author with the diligence he deserves, and will amply repay, may discover a change in his practice, arising, we can hardly doubt, from a change in the character of diseases. In the early part of his career, he has no difficulties concerning evacuations, not only in fever, but in gout, rheumatism, and most other diseases: later in life, he appears to have been more cautious. In a character equally diligent, sagacious, and decided, such a change can only be imputed to experience, acquired by the closest observation. Is it not reasonable, then, to suppose, that a practice most of us are old enough to recollect is now going into disuse, not merely from fashion, but from an actual change in the character of diseases? Is not this confirmed by the observations of one whose lessons many of us had the happiness to sit under? Dr. George Fordyce used to acknowledge, and his writings still testify, that early in his practice he was more free with the lancet than he could afterwards venture to be? Do we not actually find him prescribing Peruvian bark, with freedom and with success, for complaints in many respects similar to those which earlier in life he cured by evacuations? Let us, therefore, be equally cautious how we condemn the practice only a few years old; and, also, lest we should continue the present under a change of those causes which may affect the human frame in a different manner.

Hitherto, however, the depleting practice seems to become more and more necessary. From infancy to extreme age,—from the highest rank to poverty under many privations,—we meet with few diseases unattended with inflammation; and, for the most part, the symptoms are urgent in proportion as the causes of inanition seem greater. It is true that, in the latter cases, we are not authorised in such bold practice; but, unless we see our patient early, we find rather the sequelæ of inflammation than its active state. In the young and wealthier, the vessels resist this change longer; that is, inflammation continues longer in the adhesive stage, and, if not removed by the first bleeding, the system will readily admit a frequent repetition of that and other evacuations.

In the midst of all this, a mild epidemic catarrh has arisen very general in every part of the town, and rarely attended with severe symptoms, excepting when the glands of the throat have been inflamed.

Many reports of mitigated small-pox, *post vaccinationem*, continue to arrive from the country, and are not entirely unknown in the metropolis. They are not, however, confirmed by the reports from St. Pancras.

The termination of phthisis, as remarked in a former report, continues very frequent, and with the same peculiarities.

The number of diseases in females, which may be traced to the changes which take place at a particular period of life, obliges us to caution our younger readers to be on their guard, and carefully attend to this important climacteric.

### A METEOROLOGICAL JOURNAL,

By Messrs. William Harris and Co. 50, Holborn, London.

*From the 20th January to the 19th February, inclusive.*

| Day of Month. | Moon. | Rain gauge | Therm. | | | Barom. | | De Luc's Hygrom. | | | Wind. | | Atmospheric Variation. | | |
|---|---|---|---|---|---|---|---|---|---|---|---|---|---|---|---|
| | | | | | | | | Dry | Damp | | | | | | |
| Jan | | | | | | | | | | | | | | | |
| 20 | | | 36 | 41 | 36 | 30·00 | 30·07 | | 7 | 7 | W | W | Fine | | |
| 21 | | | 38 | 42 | 35 | 29·97 | 30·14 | | 11 | 9 | WSW | WSW | Fine | | |
| 22 | ○ | | 39 | 45 | 35 | 29·85 | 29·58 | | 12 | 12 | SSW | W va. | Fine | Rain | |
| 23 | | | 36 | 40 | 39 | 29·58 | 29·65 | | 10 | 10 | W | SW | Fine | Rain | Fine |
| 24 | | | 40 | 42 | 33 | 29·65 | 29·69 | | 9 | 9 | W | W | Rain | | Fine |
| 25 | | | 35 | 39 | 35 | 29·98 | 29·90 | | 10 | 12 | W | SW | Fine | | Rain |
| 26 | | | 35 | 42 | 37 | 29·78 | 29·54 | | 12 | 15 | W | SSW | Rain | | Clo. |
| 27 | | | 39 | 43 | 39 | 29·50 | 29·50 | | 15 | 20 | W | S | Rain | | Clo. |
| 28 | | | 40 | 43 | 34 | 29·54 | 29·64 | | 20 | 15 | W | SW | Rain | Fine | |
| 29 | ☽ | | 36 | 39 | 39 | 29·50 | 29·22 | | 15 | 15 | S | S va. | Rain | | |
| 30 | | | 41 | 43 | 35 | 29·20 | 29·14 | | 10 | 15 | W | SSW | Rain | | |
| 31 | | | 36 | 40 | 34 | 29·27 | 29·51 | | 18 | 14 | WSW | SSW | Fine | | |
| Feb | | | | | | | | | | | | | | | |
| 1 | | | 36 | 38 | 31 | 29·40 | 29·15 | | 15 | 13 | WSW | SW | Rain | Fine | |
| 2 | | | 32 | 34 | 29 | 29·04 | 29·14 | | 12 | 14 | SSE | WSW | Fog | Fine | |
| 3 | | | 30 | 37 | 29 | 29·16 | 29·25 | | 14 | 10 | S | SW | Clo. | Hail | Fine |
| 4 | | | 30 | 34 | 28 | 29·30 | 29·39 | | 7 | 10 | W | NNW | Fine | | |
| 5 | ● | | 29 | 33 | 32 | 29·54 | 29·88 | | 16 | 15 | SW | SW | Fog | Fine | |
| 6 | | | 32 | 35 | 29 | 29·95 | 30·00 | | 18 | 20 | SW | NE | Fog | Fine | Fog |
| 7 | | | 30 | 37 | 26 | 30·15 | 30·00 | | 23 | 25 | NE | N | Fog | | |
| 8 | | | 29 | 30 | 26 | 29·99 | 29·97 | | 27 | 22 | W | W | Fog | Clo. | Fog |
| 9 | | | 26 | 30 | 26 | 30·00 | 30·00 | | 25 | 22 | W | NW | Fog | | |
| 10 | | | 27 | 31 | 31 | 30·00 | 30·10 | | 22 | 20 | N | NE | Fog | | Fine |
| 11 | | | 32 | 35 | 31 | 30·10 | 30·19 | | 18 | 19 | N | N | Fine | | |
| 12 | | | 32 | 35 | 30 | 30·21 | 30·14 | | 15 | 16 | E | SE | Fine | Clo. | Fine |
| 13 | ☾ | | 30 | 35 | 30 | 30·15 | 30·15 | | 13 | 13 | S | E | Fine | | |
| 14 | | | 32 | 37 | 31 | 30·25 | 29·10 | | 12 | 12 | S | SE | Fine | | |
| 15 | | | 37 | 41 | 34 | 29·87 | 29·85 | | 12 | 17 | S | SE | Fine | Rain | Clo. |
| 16 | | | 39 | 44 | 40 | 29·90 | 29·96 | | 22 | 20 | S | SSE | Clo. | Rain | Clo. |
| 17 | | | 43 | 48 | 41 | 30·00 | 30·00 | | 25 | 20 | SE | SE | Clo. | Rain | Clo. |
| 18 | | | 44 | 48 | 42 | 29·90 | 29·88 | | 23 | 21 | S | S | Clo. | Rain | Fine |
| 19 | | | 43 | 49 | 37 | 29·90 | 29·94 | | 20 | 18 | S | NW | Fog | Rain | |

The thermometer having been frequently below the freezing-point in the month of January, the Rain-gauge has become useless.

